# Report of Cases—Thirty-Five Operations for Mastoiditis

**Published:** 1902-01

**Authors:** James M. Crawford

**Affiliations:** Atlanta, Ga.


### REPORT OF CASES—THIRTY-FIVE OPERATIONS FOR MASTOIDITIS.

BY JAMES M. CRAWFORD, M.D.,

ATLANTA, GA.

In the last six years I have performed thirty-five operations for mastoiditis. Without entering into the tiresome details of each individual case, I will present a few clinical facts and conclusions for discussion.

Of the thirty-five cases, nine were from scarlet fever, the average time being four-and-one-half weeks after scarlet fever had been pronounced; four were from chronic middle ear trouble, and the remaining twenty-two were from acute suppuration of the middle ear; fourteen were under the age of ten years and six were over forty years of age. I have operated the second time twice. In two cases I operated upon each mastoid; in seven cases I operated at night. I can readily say that I had as soon operate at night as during the day. In two cases I operated without any anaesthetic since it was out of the question to give anything of the kind, as they had bad heart trouble.

The most common cause of mastoiditis is an extension of an inflammation from the middle ear through the aditis to the antrum. I have found it occurring, as you see, more frequently from acute suppuration of the middle ear.

Primary mastoiditis is occasionally seen and may follow an exposure to cold or intraumatism, or may be a manifestation of some specific diathesis. The most prominent symptoms met with in mastoiditis, and upon which most dependence can be placed, are local tenderness over the mastoid region and a sagging of the superior posterior wall of the external auditory canal, close to the tympanic ring. We may have them combined, but either one alone often constitutes the only sign upon which the necessity of operative procedure is based. Meningitis is sometimes the result of mastoiditis, and hence a symptom not to be overlooked. The temperature cannot always be relied upon as a symptom, since I have made the operation in cases where this was not above normal.

In determining mastoid tenderness, care must be taken to be sure that the pain experienced by the patient is mastoid tenderness and not tenderness from the external auditory canal. Where there is tenderness and swelling, there is one trouble with which mastoiditis may be confounded, namely, furuncle of the external auditory canal. Most usually in cases of furuncle, the swelling is located anterially, while in mastoiditis the swelling is posterially. A very good point, too, is to press the finger backward and inward upon the mastoid just behind the insertion of the auricle. This procedure does not move or press against the canal. On examination the most tender point is situated over the antrum.

Generally, no difficulty is experienced in making the diagnosis; on the other hand, we meet with cases in which we are in doubt. I was called to a case of this character a short time ago by Dr. Huguely at Barnesville, Ga. The patient had no signs whatever of mastoiditis, but marked signs of meningitis and a running ear. Absolutely, there was no swelling over the mastoid, nor pain on pressure. The history of his case was this: About two weeks before, Dr. Huguely was called to see the patient, who was suffering with great pain in the ear. After continuing to suffer from such pain for several hours, the drum membrane ruptured and the ear began to run. Even at this time there was marked tenderness over the mastoid which lasted for about three days, when the swelling and pain gradually subsided. Marked symptoms of meningitis, however, followed this recession of pain and swelling. For this reason only I decided to operate. On entering the antrum I found a great amount of pus, but no opening into the brain cavity. This patient is now well; I feel confident that she would have died soon had not the operation been made.

The only case that I have had to end fatally was that of a man about forty years of age. He had had a discharging ear for about six weeks, had been unconscious for two-and-one-half days when I saw him first. He never regained consciousness after the operation, but lived several days and died from meningitis.

On May 14th, 1900, Mrs. E. M. S. came to my office presenting marked symptoms of acute aural catarrh of the right ear. Hoping to avert any operation I sprayed the nose and throat and inflated the tympanic cavity. She returned the next morning no better, at which time I incised the drum membrane well and found pus. When she came in the next morning I soon saw that an operation upon the mastoid would be needful. I madean arrangement to operate the next day. Upon my arrival I found that the symptoms, both pain and swelling, had disappeared. She stated to me that about 1 o'clock the night before, relief came suddenly. I could get no tenderness over the mastoid, nor did she present any other symptoms of mastoiditis. However, I operated and found a great amount of pus, also an opening into the brain cavity, the rupture of which was accountable for her relief. This patient would have died had she not had the operation made. I am in favor of early and radical operative procedure.

The instruments required in this operation are: A medium-sized scalpel, curved scissors, fixation forceps, retractors, rawhide mallet, various sized chisels, ronguer forceps with different sized blades, a small probe, sharp spoon curettes, periosteum elevators, and artery forceps. I favor chloroform as an anesthetic. The ear should be thoroughly cleansed and tamponed with bichloride gauze; the mastoid region shaved and thoroughly cleansed with bichloride solution, 1 to 1000, and covered with towels dipped into the same solution. All instruments should be sterilized by boiling, and the operator's and assistant's hands receive the necessary attention. Strict antiseptic principles should be observed throughout the operation, for we do not know when we are to enter the brain cavity.

The primary incision begins about a half an inch below the tip of the mastoid process and is carried upward close to the line of the insertion of the auricle to a level with the superior extremity of the auricle. The line of incision should be made close to the line of the insertion of the auricle so that when the flaps are retracted the superior walls of the external auditory canal are freely exposed. The incision made in this way also heals up, leaving very little deformity. Throughout the entire incision, the soft part, including the periosteum, should be divided to the bone. From about the center, and at right angles to the primary incision, a second incision about one inch is made directly backward. This second incision makes two posterior flaps, which renders it very much easier to be elevated and pushed out of the field of operation. The superior posterior flap is now elevated and pushed upward and backward, well out of the way. The anterior flap is then elevated and pushed forward until the posterior and superior margins of the external auditory canal are clearly seen. The post inferior flap is now elevated and pushed backward and downward. With blunt, curved scissors the sterno-mastoid muscle is next cleared from the tip of the process. In this procedure the scissors should be made to hug the bone very closely so as not to wound any of the large vessels of the neck.

Hemorrhage in this operation is never very great. I seldom find it necessary to tie a vessel, tortion only being necessary. A retractor is now placed behind the anterior flap and held forward by the assistant. The flaps being pushed well out of the way, the wound is sponged out by the assistant and the operator finds his anatomical landmarks.

The antrum is best located by bearing in mind its relation to the superior and posterior walls of the external auditory meatus. If a triangle is formed, one line of which is drawn horizontal with the root of the zygoma, another vertical and tangent to the posterior wall of the external auditory canal, then finish triangle with straight line; this triangle is known as post meatal triangle. This triangle lies immediately over the antrum.

Having located the antrum we proceed to enter it. The cortex is best removed by means of the mallet and chisel; a large curved chisel is used at first, being applied nearly parallel to the surface of the skull, and is made to cut away the bone in thin, broad chips. In this way we form a bony channel, the center and deepest part of which should correspond to the antrum. We know that the antrum is entered by its depth. A probe placed in the artificial opening and then placed in the external auditory canal will find both to be of the same depth. With rouguer forceps and sharp spoon curettes, the whole of the pneumatic structure of the mastoid process is obliterated, and all overhanging bone removed. The operation should be continued until sound bone is encountered in every direction. After all of the cells have been removed and the operation completed, the wound is packed with bichloride gauze. Before this, however, I found it well to place a few stitches in the wound, one stitch being placed at the superior extremity and one at the inferior extremity of the incision. The second incision is closed up entirely. I found this to give better results than to leave the wound entirely open. Bichloride gauze is then placed over the wound, on top of which is placed a piece of sterile cotton and held in place with a bandage.

In cases where the technique has been perfect it is not necessary to remove the dressing under five or six days. At each dressing, which should be about five days apart, the wound and the external auditory canal should be well irrigated with a hot bichloride solution, 1 to 5000.

Exposure of the lateral sinus is not a very dangerous occurrence, but when it does occur, it is difficult to proceed with the operation. I have had only one case where the lateral sinus was accidentally exposed. Fortunately for me in the case I have just mentioned, the operation was about completed when I undertook to ascertain the size of the opening in the cranial cavity, and I had the misfortune to lacerate the walls of the internal lateral sinus. The bleeding was immediately checked by a compress made of gauze, which was not removed for seven days, at which time there was no bleeding upon dressing the same.

These cases are particularly interesting since they are different from the old way of operating for mastoiditis when Wild's incision, followed by a poultice, was all that was done to complete the operation. We can now see how useless such an incision is in such cases.

